# Cochlear Cell Atlas of Two Laryngeal Echolocating Bats—New Evidence for the Adaptive Nervous Physiology in Constant Frequency Bat

**DOI:** 10.1111/1755-0998.14101

**Published:** 2025-03-20

**Authors:** Xue Wang, Mingyue Bao, Hui Wang, Ruyi Sun, Wentao Dai, Keping Sun, Yue Zhu, Yingting Pu, Yujia Chu, Xintong Li, Tianhui Wang, Minjie Zhang, Aiqing Lin, Jiqian Li, Jiang Feng

**Affiliations:** ^1^ College of Life Science Jilin Agricultural University Changchun China; ^2^ Jilin Provincial International Cooperation Key Laboratory for Biological Control of Agricultural Pests Changchun China; ^3^ Jilin Provincial Key Laboratory of Animal Resource Conservation and Utilization Northeast Normal University Changchun China

**Keywords:** bats, cell atlas, cochlea, neurons, snRNA‐seq

## Abstract

Bats have evolved highly adapted auditory mechanisms associated with ecological specialisation. However, there is scattered knowledge about the neurophysiological and cellular basis underlying high‐frequency hearing in echolocating bats. Herein, the total cochlear cell atlas of *Rhinolophus ferrumequinum* (constant frequency (CF) bat) and *Myotis pilosus* (frequency modulated (FM) bat) was conducted using the 10x Genomics single‐nucleus RNA sequencing method. Differences in the proportion of cochlear cell types, especially for the neural cells, were detected between these two bat species. Previously, genes upregulated in the cochlea of CF compared with FM bats, were found to be mostly related to nervous activities. After mapping to the cochlear cell atlas, we found that the upregulated genes were from neural cells, lateral wall cells and neurosensory epithelium cells. A class of specific neurons and associated functions was detected in the cochlea of *R. ferrumequinum* , revealed by cross‐species single‐cell transcriptomic analyses. Furthermore, molecular evidence for the differentiation from glial cells to neuronal cells was also uncovered in the cochlea of *R. ferrumequinum* . Overall, this study identified specific cellular molecular properties that constitute the neuroanatomical evolutionary dynamics underlying distinct echolocating types of bats and provided new molecular evidence for high‐frequency hearing of echolocating bats, promoting related studies about ecological adaptation and evolution.

## 1 Introduction

Distinctive traits distinguish species and often reflect their unique adaptive evolution. Bats have evolved remarkable echolocation ability and excellent high‐frequency hearing compared to other echolocators from rodents ( *Typhlomys chapensis* ) and whales ( *Mesoplodon densirostris* ) (Jones and Holderied 2007; He et al. 2021; Madsen et al. 2013). Among mammals, bats possess the widest range of vocalisations, spanning from below 20 kHz to over 200 kHz and have consequently developed highly specialised hearing sensitivities (Ramsier et al. 2012).

The auditory organs and associated structures in bats have undergone distinctive adaptive changes. Notably, in contrast to non‐echolocating mammals of comparable size, echolocating bats often exhibit enlarged external ears (Davies et al. 2013). This anatomical adaptation is crucial for the efficient reception of subtle echolocation calls, which in turn significantly amplifies the accuracy of their spatial orientation and prey detection. Specifically, the cochleae of laryngeal echolocating bats are notably enlarged, comprising 2.5 to 3.5 spiral turns, a stark contrast to the average of 1.75 turns in non‐echolocating fruit bats (Kössl et al. 1999). This enhanced cochlear configuration endows a refined frequency discrimination, with the basal turn being specialised for high frequencies and the apical turn for low frequencies. Additionally, the basilar membrane in the cochleae of echolocating bats is notably elongated, featuring a robust and constricted basal segment. The outer hair cells situated on this membrane display a denser packing and reduced length, complemented by shorter stereocilia (Vater and Kössl 1996). These structural features collectively enhance the echolocating bats' perception of echo signals, enabling precise echolocation in complex environments. Furthermore, different types of echolocating bats have represented distinct adaptive changes in their inner ear (Davies et al. 2013).

Generally, laryngeal echolocating bats could be categorised into two types according to their echolocation calls: frequency modulated (FM) bats and constant frequency (CF) bats (Kazial et al. 2008). Besides the frequency‐modulated components, CF bats possess a distinctive constant‐frequency element within their vocalisations (Ruczynski et al. 2007). For instance, CF bats from the *Rhinolophidae* family exhibit a notably enlarged cochlear basal turn, enabling precise inner ear tuning in response to the specialised CF echoes emitted by themselves (Davies et al. 2013; Russell et al. 2003). Furthermore, within the cochleae of CF bats, a specialised structure referred to as the auditory fovea is present on the basilar membrane (Russell and Kössl 1999). This region exhibits an expanded representation of frequencies, particularly attuned to the CF component, and represents a pronounced overexpression of neurons, thereby providing a heightened sensitivity and refined discrimination to the relevant sound signals (Schnitzler and Denzinger 2011). This cochlear structure in CF bats facilitates the compensation for Doppler shifts within the complete echo signals, a capability crucial for maintaining precise auditory perception despite the motion of targets or the bats themselves (Schnitzler and Denzinger 2011; Suga and Schlegel 1973).

In recent work by R. Benjamin on the evolution of inner ear neuroanatomy of bats and implications for echolocation, a significant difference was observed in spiral ganglion structures between *Yinpterochiroptera* (containing CF bats) and *Yangochiroptera* (mainly containing FM bats) (Sulser et al. 2022). In *Yinpterochiroptera*, the spiral ganglion is encased in a protective Rosenthal's canal, while *Yangochiroptera* displays a wall‐less Rosenthal's canal, suggesting that the FM bats have developed an enlarged spiral ganglion liberated from the constraints of bony canal porosity, which allows for a larger ganglion with more neurons, higher innervation density and denser clustering of cochlear nerve fascicles. This finding not only expands our comprehension of the bat inner ear's structural nuances but also offers significant clues for deciphering the evolutionary underpinnings of their echolocation capabilities.

In addition to the revolutionary progress in the neurophysiology and morphology of the cochlea, the comparative molecular biological investigations into the cochleae of different types of echolocating bats have unveiled findings that are equally as exhilarating (Wang et al. 2018). Previous studies provided strong evidence for the adaptive evolution of several auditory genes, including *Prestin*, *KCNQ4* and *Tmc1*, which are implicated in the high‐frequency hearing abilities of echolocating bats (Li et al. 2008, 2010; Davies et al. 2012; Liu et al. 2012). This evolutionary adaptation is crucial for the development and refinement of the bats' acute auditory perception at high frequencies. Compared with FM bats, the cochleae of CF bats exhibited a number of upregulated genes and were closely related to neuronal activity, which may be caused by a large number of different types of neural cells in the cochlea of CF bats (Wang et al. 2018). However, the exact source cell types from which these genes come, the complex interactions among these cells and the biological functions they perform are still unclear. Previous findings consistently indicated that the complexity and specialisation of the bat auditory system are reflected in its structure and also fine molecular regulation (Neuweiler 2003; Teeling et al. 2016; Liu et al. 2021). We are eager to correlate the morphological differences with the molecular adaptations to identify the possible differences in the types, numbers and regulation mechanisms of neural cells in the cochleae of the two types of echolocating bats. At the same time, we will also focus on the differences in the evolution of the auditory system in different bat species to better understand the relationship between biodiversity and ecological adaptation. With the development of molecular biology and the improvement of sequencing technology, single‐cell/single‐nucleus RNA sequencing (scRNA‐seq/snRNA‐seq) is an unparalleled method for assessing the gene expression profiles, cellular diversity and heterogeneity of tissues at the single‐cell level. More and more evidence shows that scRNA‐seq/snRNA‐seq has great advantages in revealing cell types in aim tissues, especially in identifying different cell populations across species, and serves as an important bridge between genes and biological phenotypes (Jean et al. 2023; Li et al. 2020).

Herein, the snRNA‐seq method was used to systematically characterise various cell types and gene expression patterns in the cochleae of two species. We focused on differences in the quantity and diversity of neural cells in the cochleae between CF and FM bats, integrating histological sections to verify the differences in cochlear structure and cellular physiology. The results of this study could more accurately uncover the key cell types and genes related to auditory adaptations in different echolocating bats, clarify the potential differentiation relationship between key neural cells, and connect the structural and cellular molecular data of echolocating bats, which is of great significance to fully reveal the cellular and molecular mechanisms underlying high‐frequency hearing in echolocating bats.

## 2 Materials and Methods

### 2.1 Sample Collection

In July 2021, individuals of *Rhinolophus ferrumequinum* and *Myotis pilosus* were successfully captured in Fangshan District, Beijing, China (115°59′ N, 39°43′ E). To avoid any influence of sex‐related differences, only males were selected for the study. The bats were collected using mist nets when they returned to their roost site after predation. Each individual bat was placed into a sterilised cloth bag. Three individuals of each bat species were rapidly euthanised by cervical dislocation, and their bilateral cochleae were collected and transferred to RNase‐free PCR tubes. Tissue samples were frozen immediately in liquid nitrogen and stored at −80 °C for later use.

### 2.2 Preparation of Cochlea Single‐Cell Nuclei Suspensions

Given the diverse array of irregularly shaped neuronal cells in the cochlea, snRNA‐seq was preferred over scRNA‐seq, which is less effective at capturing these cells. Additionally, snRNA‐seq is highly effective in capturing rare or hard‐to‐isolate cell types and provides a more comprehensive representation of cell types, particularly in complex tissues like the bat cochlea, which are challenging to dissociate. Therefore, snRNA‐seq was used in this study. The cochleae from each bat species were cut into small pieces. The remaining liquid was blotted on the surface of the tissue with absorbent paper, and placed on a 10‐cm disposable sterile Petri dish. The tissue was chopped into 1–2 mm^3^ pieces on ice. The pieces were added to the pre‐chilled nucleus wash buffer and gently moved in and out five times to facilitate the extraction of nuclei. Tissue homogenates were filtered through a 70‐μm cell sieve to obtain approximately 1 mL of filtrate, and iodixanol was added to prepare a gradient solution. After centrifugation, the supernatant was collected, and the nuclear suspension was filtered through a 40‐μm cell sieve to ensure a homogeneous preparation. Each cell pellet was resuspended in buffer, stained with trypan blue and observed under a microscope to calculate cell concentration. The nuclear suspension with a cell concentration of 700–1200 cell/μL was used for future sequencing.

### 2.3 cDNA Library Preparation and Single Nucleus RNA‐Sequencing

Nuclear suspensions were loaded on a 10x Genomics GemCode Single‐cell instrument that generates single‐cell Gel Bead‐In‐Emulsion (GEMs). Upon dissolution of the Gel Bead in a GEM, primers containing (i) an Illumina R1 sequence (read 1 sequencing primer), (ii) a 16 nt 10x Barcode, (iii) a 10 nt Unique Molecular Identifier (UMI), and (iv) a poly‐dT primer sequence were released and mixed with cell lysate and Master Mix. Barcoded, full‐length cDNAs were then reverse‐transcribed from poly‐adenylated mRNA. Silane magnetic beads were used to remove leftover biochemical reagents and primers from the post‐GEM reaction mixture. Full‐length, barcoded cDNAs were then amplified by PCR to generate sufficient mass for library construction. R1 (read 1 primer sequence) was added to the molecules during GEM incubation. The final libraries contained the P5 and P7 primers used in Illumina bridge amplification. Libraries were generated and sequenced from the cDNAs with Chromium Next GEM Single Cell 3′ Reagent Kits v3.1.

### 2.4 Bioinformatic Analysis

Data quality control and gene expression quantification 10x Genomics Cell Ranger software (version 3.1.0) was used to convert raw BCL files to FASTQ files, alignment and counts quantification (Zheng et al. 2017). The reads were then mapped to the *R. ferrumequinum* genome. Subsequently, we executed similar conversion, alignment and quantification steps for the raw BCL files of *M. pilosus* , aligning the reads to the full‐length transcriptome of *M. pilosus* (Wang et al. 2022). The raw snRNA‐seq data for both bat species have been deposited in the NCBI Sequence Read Archive database with accession numbers SRR31753503 for *R. ferrumequinum* and SRR31753632 for *M. pilosus* .

#### 2.4.1 Cell Clustering

The cell by gene matrices for each bat species were individually imported to Seurat version 3.1.1 for downstream analysis (Butler et al. 2018). Expression Quality Control (QC) cells with an unusually high number of UMIs (≥ 3000) or mitochondrial gene percent (≥ 10%) were filtered out. We also excluded cells with fewer than 200 or more than 1500 genes detected. Additionally, doublet GEMs were filtered out as well. Then, the cell‐by‐gene matrices for each sample were individually imported to Seurat version 3.1.1 for downstream analysis. Briefly, Seurat embeds cells in a shared‐nearest neighbour (SNN) graph, with edges drawn between cells via similar gene expression patterns. To partition this graph into highly interconnected quasi‐cliques or communities, we first constructed the SNN graph based on the euclidean distance in PCA space and refined the edge weights between any two cells based on the shared overlap in their local neighbourhoods (Jaccard distance). We then cluster cells using the Louvain method to maximise modularity (Rotta and Noack 2011). For visualisation of clusters, *t*‐distributed Stochastic Neighbor Embedding (t‐SNE) were generated using the same PCs (Laurens and Hinton 2008).

#### 2.4.2 Differentially Expressed Genes Analysis

In order to analyse the characteristics of the transcriptional regulation patterns of individual cell subclusters, and to further screen the gene markers specifically expressed from each subcluster, we used the likelihood‐ratio test to find differentially expressed genes (DEGs) for a single cluster, compared to all other cells. Then, Seurat's bimod likelihood ratio statistical test was used to perform differential gene expression analysis on different cell clusters, and each cluster's upregulated genes were screened. Genes had to be at least 1.28‐fold overexpressed in the target cluster. Second, genes had to be expressed in more than 25% of the cells belonging to the target cluster. Third, the adjusted *p* value (after multiple test correction using the Benjamini–Hochberg method) was less than 0.01. To elucidate the biological processes mediated by key upregulated genes in each cochlear cell type of *R. ferrumequinum* and *M. pilosus* , downstream functional classification was performed by the integrated localisation of GO (Harris et al. 2004) and KEGG pathway databases (Ogata et al. 1999). All *p* values underwent multiple testing correction using the Benjamini‐Hochberg method, with a false discovery rate (FDR) cut‐off of 0.01, namely *q* values < 0.01.

#### 2.4.3 Weighted Gene Co‐Expression Network Analysis (WGCNA)

To enhance the accuracy of identity allocation, WGCNA was performed on the single‐cell expression matrices of *R. ferrumequinum* and *M. pilosus*, respectively. Co‐expression networks were constructed using the WGCNA (version 1.47) package in R (Langfelder and Horvath 2008). The specific methods are detailed on GitHub (GitHub‐Bioinformatics‐rookie/WGCNA). For *R. ferrumequinum* , the expression values of 19,093 genes were imported into WGCNA, and co‐expression modules were constructed using the default settings of the blockwise Modules function. After 15 iterations, the genes were grouped into 82 initial modules, which were subsequently consolidated into 21 core modules. Similarly, for *M. pilosus* , the expression values of 12,786 genes were imported into WGCNA, and the same blockwise Modules function was applied with default settings. After 15 iterations, the genes were divided into 67 initial modules, which were further consolidated into 19 core modules. For genes in the selected module, GO and KEGG pathway enrichment were conducted to analyse the biological functions according to the method of Section 2.4.2.

#### 2.4.4 Cell‐Chat Signalling Network Analysis

Cell‐cell communication analysis was performed on the single‐cell data of *R. ferrumequinum* and *M. pilosus* , using CellChat (version 1.6.1), respectively (Jin et al. 2021). The detailed methods are available on GitHub (GitHub‐jinworks/CellChat: R toolkit for inference, visualisation, and analysis of cell‐cell communication from single‐cell and spatial transcriptomics). For each species, the normalised expression matrices, cell grouping information and receptor–ligand database were imported into CellChat to construct CellChat objects. By integrating gene expression data with known interactions between signalling ligands, receptors and their cofactors, we assessed the probability of cell‐cell communication based on the single‐cell gene expression matrices for each species and predicted cell–cell communication accordingly.

#### 2.4.5 Pseudo‐Time Trajectory Analysis

To observe the differentiation relationships among different cell clusters, Monocle (version 2.0) was used to construct a single‐cell pseudo‐time differentiation trajectory (Cao et al. 2019). After annotating cell types in Seurat and constructing cell developmental trajectories according to unsupervised analysis methods, the reverse graph embedding ‘DDRTree’ algorithm was used to dimensionalise the data after downscaling and aligning cells in pseudo‐time. The cell trajectory plots and heatmap visualisation were performed based on the feature values of pseudo‐time, cell type and specified genes. The genes were preserved and analysed for functional enrichment, respectively.

### 2.5 Integrative Analysis of snRNA‐Seq and Bulk RNA‐Seq Data

Previous studies have identified a substantial number of upregulated genes associated with neural activity in the cochlea of CF bats compared to FM bats, called CF‐highly expressed genes (Wang et al. 2018). However, the specific cellular origins of these highly expressed genes remain unclear. Consequently, this study mapped 2144 and 1851 upregulated genes that were identified from the bulk RNA‐seq data of two CF versus FM comparative groups, namely *Rhinolophus sinicus* versus *Taphozous melanopogon* (CF1 vs. FM) and *Aselliscus stoliczkanus* versus *T. melanopogon* (CF2 vs. FM), into the new cochlear single‐cell transcriptome data of CF bats, namely *R. ferrumequinum* , to uncover the key cellular types responsible for the unique neural activity in CF bats. First, we conducted an overlap analysis between the pathways enriched by CF‐highly expressed genes and the pathways enriched by upregulated genes in each cell type of *R. ferrumequinum* . Second, we mapped all CF‐highly expressed genes to the RNA expressed in each cell type of *R. ferrumequinum* . By mapping these upregulated genes and their associated signalling pathways onto the single‐cell data of *R. ferrumequinum* , we aimed to elucidate the cellular sources of differentially expressed genes detected at the transcriptome level in previous bulk RNA‐seq data.

### 2.6 Haematoxylin and Eosin Staining (HE) of Cochlea

To clearly compare the cochlear structures of two species, a haematoxylin and eosin staining experiment of the cochlea was conducted. Cochlea tissue of two bat species was fixed in 10% neutral buffered formalin for 24 h (Cardiff et al. 2014), dehydrated through a graded series of ethanol and then cleared in xylene prior to embedding in paraffin wax. Sections of 5‐μm thickness were cut using a microtome, mounted on glass slides and air‐dried at room temperature. For HE staining, the sections were first deparaffinised in xylene and rehydrated through a series of graded ethanol solutions. Haematoxylin was applied for 5 min to stain the cell nuclei, followed by a rinse in running tap water. The sections were then differentiated in acid alcohol, rinsed in tap water and stained with eosin for 2 min to colour the cytoplasm and extracellular matrix. After eosin staining, the slides were dehydrated through increasing concentrations of ethanol and cleared in xylene before being cover slipped with a synthetic mounting medium. The stained sections were examined using a light microscope to evaluate the cellular architecture and tissue morphology.

### 2.7 Cross‐Species Analysis of CF and FM Bats

To thoroughly compare the cellular types and functional differences between *R. ferrumequinum* and *M. pilosus* , with a particular focus on the differences in neural cells between these two bat species, a cell gene expression matrix based on homologous genes from both bat species was constructed. The one‐to‐one orthologs of the two species were retained and a total of 8607 homologous genes were identified. In detail, reference genomes of the two bat species were obtained, and BLASTP was used to perform sequence alignment with a specified threshold (*e*‐value < 1e‐5), then one‐to‐one orthologs with bidirectional best hits were selected for subsequent analysis. Based on the one‐to‐one orthologs between the two bat species, the gene expression matrices were merged. The merged matrices were then subjected to PCA for dimensionality reduction and clustering using the R package Seurat (Butler et al. 2018). Finally, t‐SNE was used to visualise the cell clusters, and a cross‐species cell atlas of cell clusters was constructed (Zhang et al. 2021).

## 3 Results

### 3.1 Cochlea Atlases of *R. ferrumequinum* and *M. pilosus*

**FIGURE 1:**
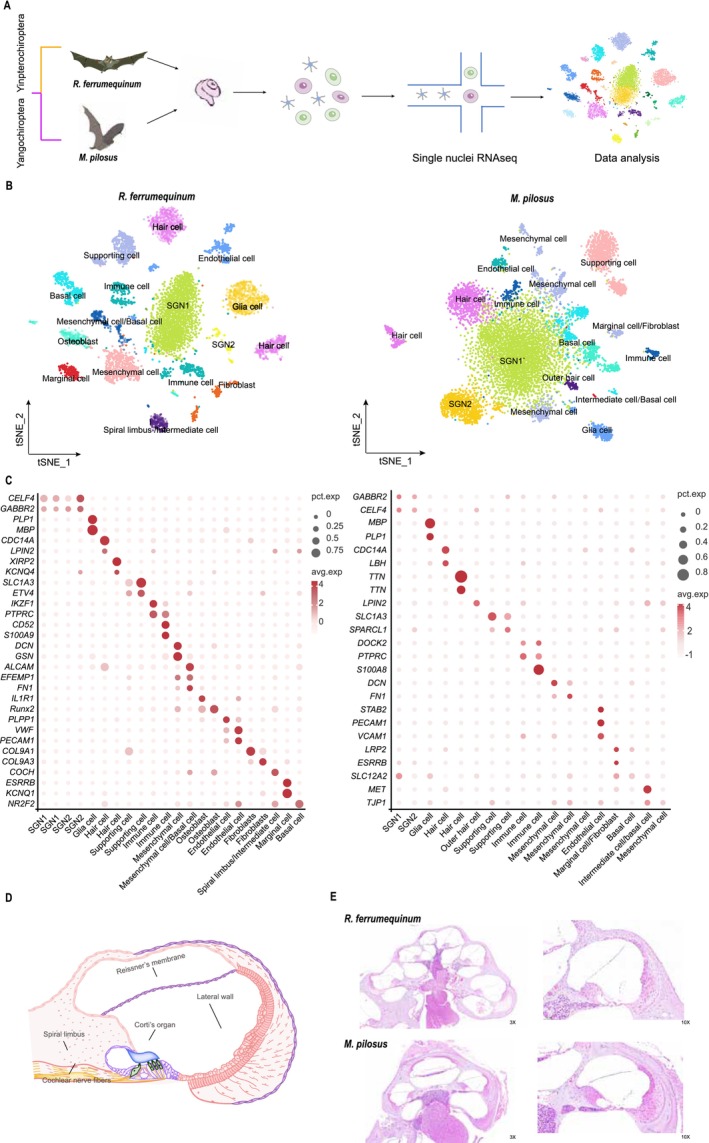
Cochlear cell atlases of *Rhinolophus ferrumequinum* and *Myotis pilosus*. (A) Experimental workflow of single‐nucleus RNA‐seq of *R. ferrumequinum* and *M. pilosus* cochleae. (B) t‐SNE illustrations of cochlear cell types in *R. ferrumequinum* and *M. pilosus* , respectively. (C) Average expression of representative marker genes in every cochlear cell type for these two bat species. (D) Schematic representation of bat cochlea structure. (E) Tissue architecture with the major sectioning planes used for HE experiments.

After 10x Genomics snRNA‐seq and stringent quality control (Figure 1A), there were 7876 single nuclei with a total of 19,093 genes, and 9703 single nuclei containing 12,786 genes were detected in the cochleae of *R. ferrumequinum* and *M. pilosus* , respectively (Figures 1 and S1A,B). By integrating the results of marker gene identification, functional enrichment analysis of highly expressed genes within each cluster, and WGCNA (Figure S2), 14 distinct cell types were identified in the cochlea of *R. ferrumequinum* : spiral ganglion neurons 1 (SGN1), spiral ganglion neurons 2 (SGN2), glial cell, hair cell, supporting cell, endothelial cell, immune cell, mesenchymal cell, fibroblast, marginal cell, basal cell, mesenchymal cell/basal cell, spiral limbus/intermediate cell, osteoblast.13 distinct cell types were identified in the cochlea of *M. pilosus* : SGN1, SGN2, glial cell, hair cell, outer hair cell, supporting cell, endothelial cell, immune cell, mesenchymal cell, marginal cell/fibroblast, basal cell, mesenchymal cell, intermediate cell/basal cell (Figure 1B,C). The t‐SNE visualisations of the cell types for *R. ferrumequinum* and *M. pilosus* were shown in Figure 1B. Figure 1D,E illustrates the schematic diagram of the bat's cochlear structure and the sectional view of cochlear tissue, respectively. According to the expression patterns of upregulated genes, candidate marker genes were identified for every cell in the cochlea of the bat (Figure S1C,D).

**FIGURE 2:**
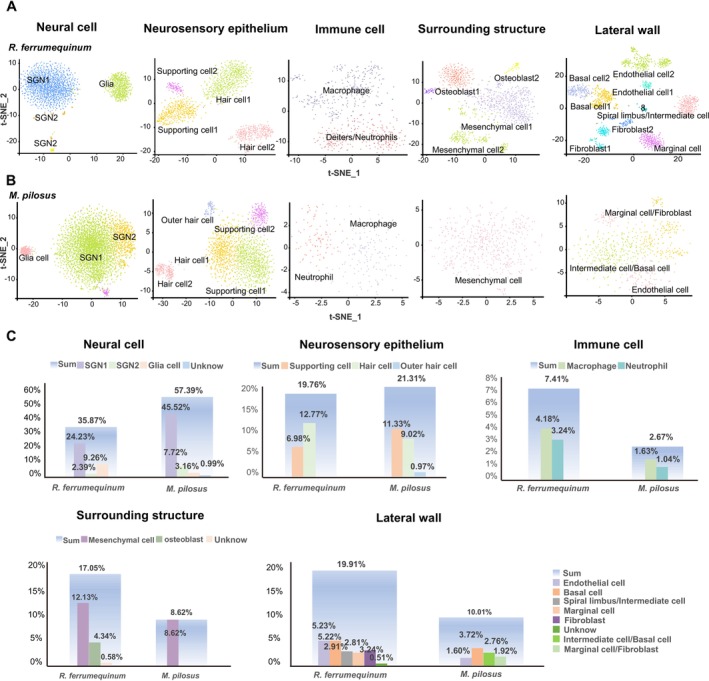
Transcriptomic characteristics of cochlear cells in *R. ferrumequinum* and *M. pilosus* . (A) t‐SNE plots of the cochlear cell types in *R. ferrumequinum* . (B) t‐SNE plots of the cochlear cell types in *M. pilosus*. (C) Bar plots showing the proportions of each cell type in cochleae of these two bat species. The proportional differences in each cell group between the two bat species are not statistically significant (*p* > 0.05).

For a better comparison of cell types between *R. ferrumequinum* and *M. pilosus* , all cochlear cells could be divided into five groups based on the cells clustering results and their spatial position (Figure 2A,B), including groups of neural cells, neurosensory epithelium cells, immune cells, surrounding structure cells and lateral wall cells. More than 200 marker genes were used for cell type identification, and detailed information on these marker genes could be found in Table S1. In detail, the neural cell group consisted of SGNs (mainly identified by *CELF4* and *GABBR2*) (Hanada et al. 2018; Li et al. 2020) and glial cells (mainly identified by *MBP* and *PLP1*) (Kim et al. 2017; Wan and Corfas 2017; Xu et al. 2022); the neurosensory epithelium cell group consisted of supporting cells (SCs, mainly identified by *SLC1A3, SPARCL1* and *ETV4*) (Shim et al. 2005; Hayashi et al. 2008; Mansour et al. 2013; Burns et al. 2015; Kubota et al. 2021) and hair cells (HCs, mainly identified by *CDC14A, XIRP2*, *Kcnq4* and *LBH*) (Scheffer et al. 2015; Imtiaz et al. 2018; Gu et al. 2021; Giffen et al. 2024); the immune cell group (mainly identified by *DOCK2, PTPRC, IKZF1, CD52, S100A9* and *S100A8*) (Clausen et al. 1999; Vandal et al. 2003; Xie et al. 2015; Hirose et al. 2017; Rai et al. 2020); the lateral wall cell group characterised by marginal cells (mainly identified by *ESRRB, KCNQ1* and *LRP2*) (Korrapati et al. 2019; Taukulis et al. 2021; Xu et al. 2022), basal cells (mainly identified by *NR2F2* and *MET*) (Korrapati et al. 2019), spiral limbus cells (mainly identified by *CALD1* and *COCH*) (Robertson et al. 2001; Xu et al. 2022; Hosoya et al. 2023), fibroblasts (mainly identified by *COL9A1* and *COL9A3*) and endothelial cells (mainly identified by *VWF, STAB2* and *PECAM1*) (Browning et al. 2005; Shi and Nuttall 2007; Usami et al. 2008). The remaining cells were classified into the surrounding structures cell group, consisting of mesenchymal cells (with *DCN, GSN* and *FN1* expression) (Buechler et al. 2021; Janesick et al. 2021) and osteoblast cells (with *IL1R1* and *Runx2* expression) (Jean et al. 2023; Qin et al. 2023).

Specifically, the proportion of cochlear neural cells in *M. pilosus* is 57% higher than the proportion of 35% in *R. ferrumequinum* (*p* > 0.05; Figure 2C). Furthermore, osteoblasts and fibroblasts were exactly identified in the cochlea of *R. ferrumequinum* , indicating a species‐specific cellular composition. Additionally, there are differences in the proportions of immune cells, surrounding structure cells and lateral wall cells between these two bat species (however, *p* > 0.05), while similar proportions of neurosensory epithelium were detected. These findings provide detailed insights into the distribution of cell types in CF and FM bat species.

### 3.2 The Cellular Locations of Highly Expressed Cochlear Genes in CF Bats Compared to FM Bats

**FIGURE 3:**
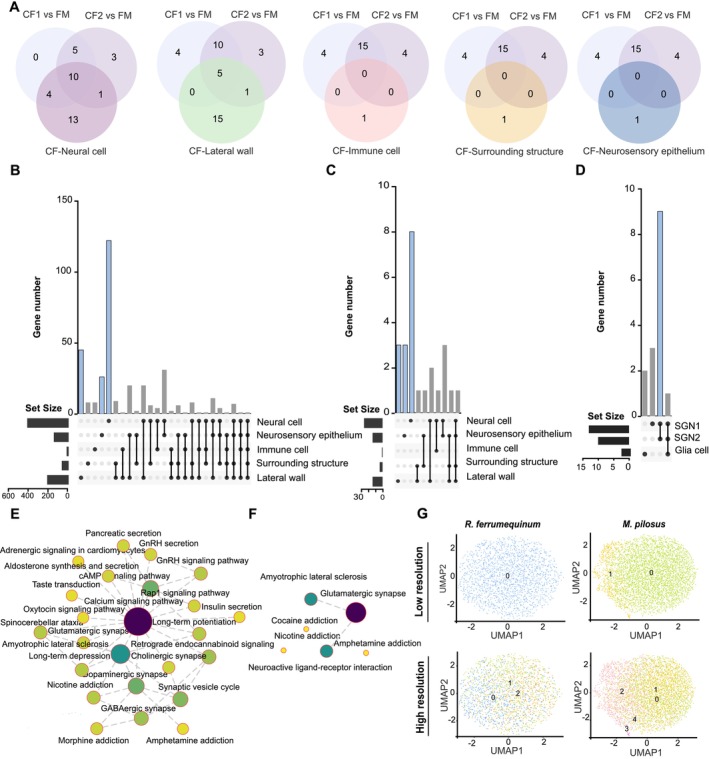
The cellular localization of highly expressed genes in the cochlea of constant frequency (CF) bats compared with frequency modulated (FM) bats. (A) Venn diagrams of the pathways significantly enriched by upregulated genes in CF bats (CF1 for *R. sinicus* and CF2 for *A. stoliczkanus* ) compared with FM bat ( *T. melanopogon* ), and also the pathways significantly enriched by upregulated genes in every cochlear cell type of CF bat ( *R. ferrumequinum* ). (B) UpSet plots display the distribution of upregulated genes in CF bats (as mentioned above) compared to FM bat ( *T. melanopogon* ) across cellular clusters in the snRNA‐seq data ( *R. ferrumequinum* ). (C) The cellular distribution of upregulated genes involved in nervous activity related pathways obtained by two CF vs. FM comparisons. (D) The localization of upregulated genes (involved in neural nervous activity related pathways obtained by two CF vs. FM comparisons) in neural cell types. (E) Network of pathways which were significantly enriched by upregulated genes detected in neural cells from *R. ferrumequinum* (*q* < 0.01). (F) Network of pathways which were significantly enriched by upregulated genes detected in neural cells from *M. pilosus* (*q* < 0.05). (Only one pathway remains significant at *q* < 0.01). (G) UMAP indicates the heterogeneity of SGN1 cell type in *R. ferrumequinum* and *M. pilosus* at different resolutions.

Previously, a number of upregulated genes related to nervous system activity were identified in the cochleae of CF bats compared with FM bats. Integrated analyses of snRNA‐seq data and bulk RNA‐seq data were performed to confirm the cells that expressed those upregulated genes above. Initially, the pathways enriched by upregulated genes in CF bats were compared with pathways enriched by upregulated genes specific to various cell types in *R. ferrumequinum* (Figure S3 and Table S2), indicating that numerous similar pathways were identified from neural cells and lateral wall cells (Figure 3A). Subsequently, all the 1365 upregulated genes in the cochlea from the CF bat were mapped to the cochlear cell atlas of *R. ferrumequinum*, indicating that those genes were mainly located in neural cells, neurosensory epithelium cells and lateral wall cells (Figure 3B). These results indicated that neural cells, neurosensory epithelium cells and lateral wall cells may play important roles in auditory perception for CF bats through specific gene regulation and associated nervous system‐related pathways.

In particular, we focused on the genes that were highly expressed in the cochlea of CF bats and related to nervous system activity, as identified in a previous study. These genes were mostly located in the neural cells of *R. ferrumequinum* (Figure 3C), specifically in the SGN1 and SGN2 (Figure 3D). Subsequently, genes with high expression levels in both snRNA‐seq data and bulk RNA‐seq data were predominantly mapped to SGN1 and SGN2 of *R. ferrumequinum* , as illustrated by four representative genes in Figure S4. These results indicated that SGN1 and SGN2 may function differently in CF bats compared with FM bats, reflected in the differential expression of genes. Downstream KEGG network analyses of the highly expressed genes in SGN1 and SGN2 from CF and FM bats, respectively, indicated an abundance and interactive array of physiological processes within the cochlea of *R. ferrumequinum* , with the calcium signalling pathway (ko04020) identified as the central signalling pathway (Figure 3E). There were only six pathways that were significantly enriched by highly expressed genes in SGN1 and SGN2 of *M. pilosus* , with the glutamatergic synapse (ko04724) as the core pathway (Figure 3F), indicating an obvious difference in neurobiological characteristics and signalling pathways within the cochleae between the two bat species. Furthermore, the SGN1 subclasses of *R. ferrumequinum* exhibited a more homogeneous distribution, while the SGN2 subclasses displayed enhanced diversity compared to those found in *M. pilosus* (as illustrated in Figures 3G and S5).

### 3.3 Cross‐Species Analysis of CF and FM Bats

**FIGURE 4:**
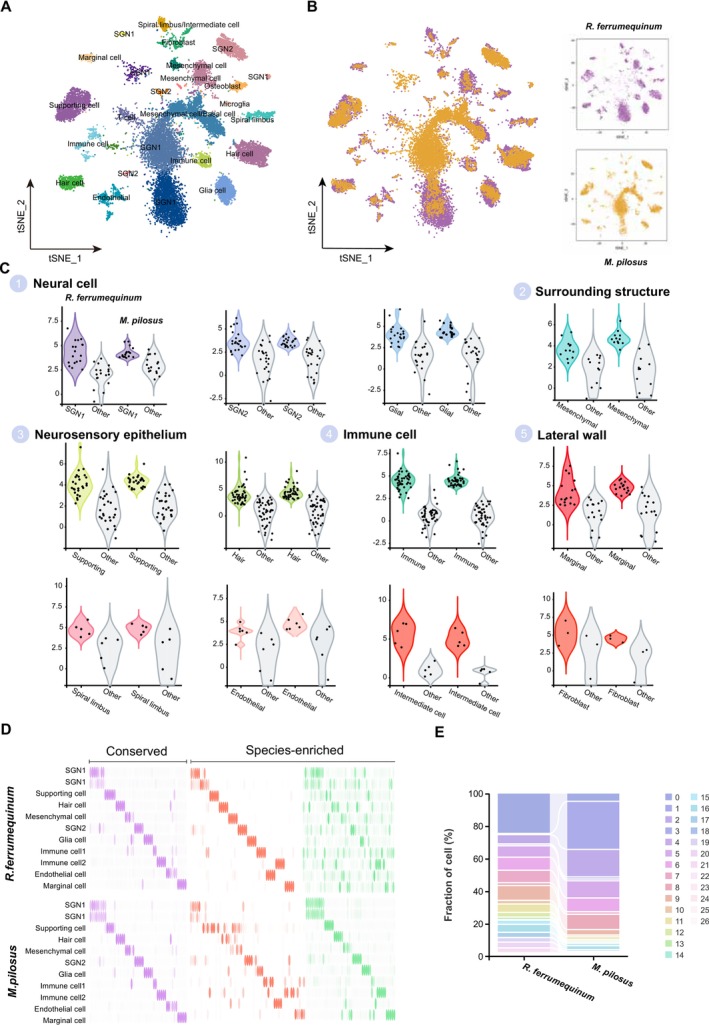
Cross‐species transcriptome comparison of cochleae at single‐cell levels between *R. ferrumequinum* and *M. pilosus* . (A) t‐SNE plot of cell atlas from cross‐species comparison results of *R. ferrumequinum* and *M. pilosus* , labelled with cell types. (B) t‐SNE visualisation of cross‐species cochlear cells based on 8607 orthologous genes. Cells from *R. ferrumequinum* and *M. pilosus* are shown in purple and orange, respectively. (C) Examples of highly similar neural cells, surrounding structures, neurosensory epithelium, immune cell and lateral wall cell types across species. Violin plots show the normalised expression of the top shared orthologous genes (FC > 1.28) in each cell type, compared to the average expression of the same genes in all other cell types. (D) Heatmap showing expression of conserved (52 genes) and species‐enriched (107 genes) DEGs, ordered by cell type and species. (E) Proportion of every cell cluster for each species. Cell Types corresponding to these 26 cell clusters were listed in Table S3.

A cross‐species cell atlas (Figure 4A,B) was successfully constructed based on 8607 orthologous genes, and 26 distinct cochlear cellular clusters were recognised for these two bat species (Figure 4E; Table S3). Important cell types were identified using the same methods as described, including SGNs, supporting cells, hair cells, mesenchymal cells and glial cells. Additionally, cross‐species analysis further confirmed the basal and mesenchymal cell types for *M. pilosus*. In‐depth comparative analysis of the cellular atlases for these two bat species revealed a high degree of transcriptional similarity at the broad cell type level, along with specific co‐expression patterns among orthologous genes (Figure 4C,D). In detail, SGNs expressed similar genes, including *PCLO, FSTL5, Fgf14*, *GRIK2* and *snap25b*. Hair cells were characterised by the expression of genes such as *CSPP1*, *CDC14A* and *Zdhhc1*. Within the immune cell, similar expression patterns were observed for genes like *COTL1*, *UBA52* and *RPS25*, highlighting a core set of immunological determinants (Table S4). These revealed conservative properties across cell types and gene expression patterns between *R. ferrumequinum* and *M. pilosus* .

**FIGURE 5:**
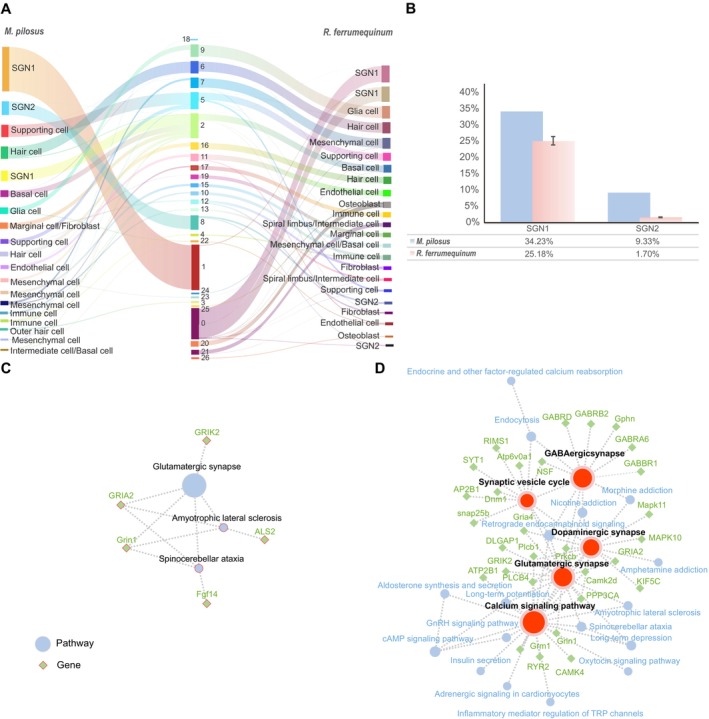
Cross‐species comparison of cochlear cells. (A) Cross‐species pairwise comparisons of cochlear cell types showing the cells flow between *R. ferrumequinum* and *M. pilosus*. (B) The proportions of SGN1 and SGN2 in the cochlea of *R. ferrumequinum* and *M. pilosus* , respectively. (C) Network of pathways significantly enriched by upregulated genes in SGN1 of *M. pilosus* (*q* < 0.01). (D) Network of pathways significantly enriched by upregulated genes in SGN1 of *R. ferrumequinum* (*q* < 0.01).

Although the cochlear cell clusters of these two bat species demonstrated a notable integration revealed by tSNE plots, species‐related differences were also represented, indicating distinct biological characteristics inherent to each species. Specifically, SGN1 (Clusters 0), osteoblasts (Clusters 20), marginal cells (Clusters 19) and spiral limbus cells (Clusters 21) were dominant in *R. ferrumequinum* , whereas SGN1 (Cluster 1) and SGN2 (Clusters 8) were dominant in *M. pilosus* . These species‐specific cell clusters were clearly illustrated in Figure 5A. The cross‐species Sankey diagrams (Figure S6) showed distinct interspecies differences in the SGNs within the neural cells, osteoblasts within the surrounding structures and spiral limbus cells within the lateral wall cells. However, no significant disparities were noted within the neurosensory epithelium and immune cell clusters, indicating a high degree of conservation in these cell clusters. According to the results of the Cross‐Species analysis, the proportion of neural cells in the cochlea of *M. pilosus* is relatively higher compared to *R. ferrumequinum* (Figure 5B). In general, similar cellular gene expression patterns in specific cell types were identified across species. Nonetheless, Figure 5A clearly illustrated that the cochlear SGN1 of *R. ferrumequinum* (designated as Cluster 0) and *M. pilosus* (designated as Cluster 1) were not intermingled, indicating substantial divergence in SGN1 between these two bat species. The pronounced disparities observed in neuronal cells between *R. ferrumequinum* and *M. pilosus* were indicative of significant interspecies heterogeneity in SGN1 cells at the single‐cell level, a finding underscored by our single‐cell resolution data. To characterise the molecular fingerprints of echolocation bat‐specific neural cell types (SGN1), 233 highly expressed genes were detected in the specific SGN1of *R. ferrumequinum* . These genes were significantly enriched in postsynapse (GO: 0098794) and postsynaptic specialisation (GO: 0099572) (Table S5). In addition, SGN1 marker genes, including *MAP2, snap25b* and *PCLO*, were contained within these 233 highly expressed genes and also the top‐ranked genes in the specific SGN1 of *R. ferrumequinum* . KEGG pathway analyses have demonstrated unique signal transduction processes that relate to diverse physiological functions and behaviours in these two bat species (Figure 5C,D). For the species‐specific neurons (SGN1) of *R. ferrumequinum* , abundant pathways were significantly enriched by highly expressed genes, including glutamatergic synapse (ko04724), dopaminergic synapse (ko04728) and GABAergic synapse (ko04727), as well as the calcium signalling pathway (ko04020) and synaptic vesicle cycle (ko04721). Notably, GABA_A_ and GABA_B_ receptors participated in the GABAergic synapse pathways, which may play a pivotal role in neurotransmitter transmission. For the *M. pilosus* ‐specific SGN1, only 26 genes were detected to be highly expressed in it and significantly enriched in amyotrophic lateral sclerosis (ko05014), spinocerebellar ataxia (ko05017) and the glutamatergic synapse (ko04724) (Figure 5D).

### 3.4 Key Interactions Between SGN and Glial Cells

**FIGURE 6:**
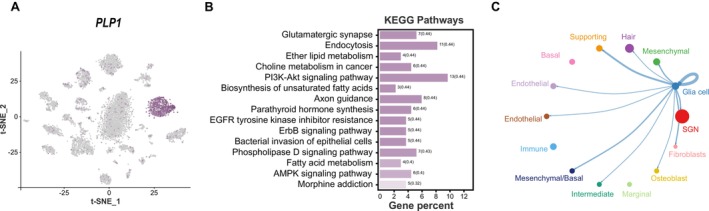
Cell communication network in the cochlea of *R. ferrumequinum* . (A) t‐SNE plot of *PLP1* gene expressed in cochlear cells. (B) The top 15 pathways significantly enriched by upregulated genes in glial cells (*p* < 0.05) (No pathway remains significant at *q* < 0.05). (C) Interactions among glia cell and other cell types.

Within the cochlea of the *R. ferrumequinum* , a robust upregulation of the neural progenitor cell marker *PLP1* is observed specifically within the glial cell population, as illustrated in Figure 6A. We found that highly expressed genes in glial cells were significantly enriched in signalling pathways related to cell differentiation, including the ErbB signalling pathway, EGFR tyrosine kinase inhibitor resistance and the PI3K‐Akt signalling pathways (Figure 6B). Furthermore, cell–cell interactions have uncovered a definitive interplay between SGN and glial cells, characterised by the engagement of Nrg1‐Erbb4 signalling and the reciprocal binding of Ncam1‐Ncam1 and Ncam1‐Ncam2 interactions (Figure 6C). These results collectively indicate an intricate network among SGN and glial cells within the cochlea of *R. ferrumequinum* .

**FIGURE 7:**
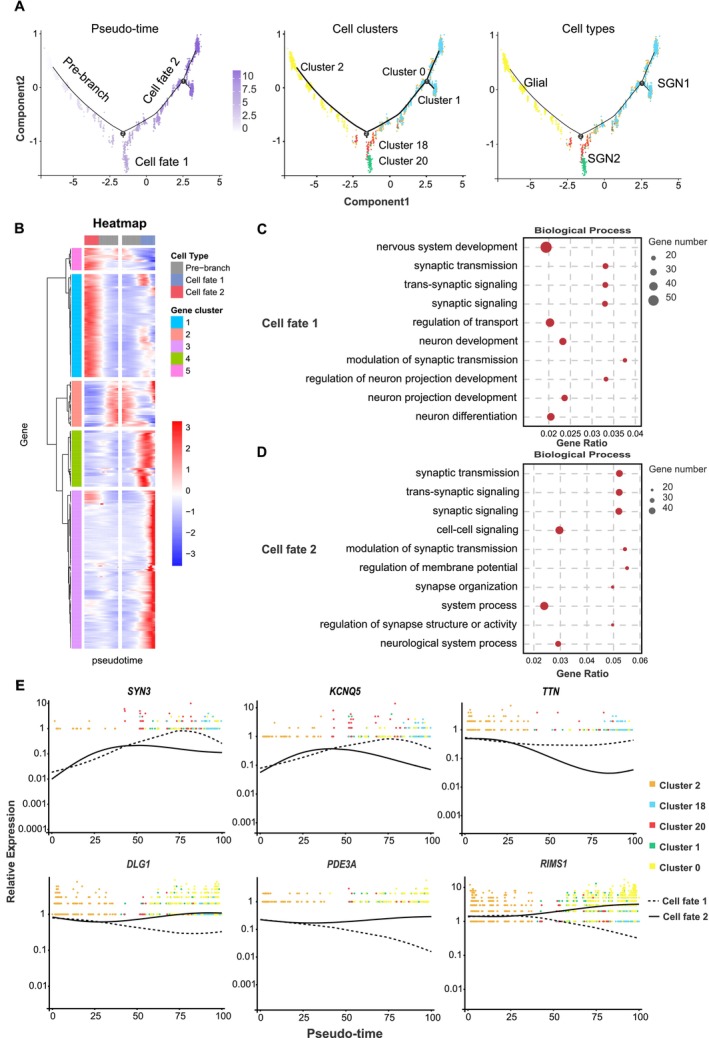
Reconstruction of the differentiation trajectory of neural cells. (A) Differentiation trajectory of neural cells with different mappings: Pseudo‐time, cell clusters and cell types. Three cell trajectories are shown in these graphs: The pre‐branch, cell fate 1 and cell fate 2. (B) Heatmap showing simulated gene expression dynamics in neural cells during cell transitions from the pre‐branch to cell fate 1 or 2. Gene sets with similar expression patterns are clustered. (C, D) The top 10 GO terms significantly enriched by differentiation genes identified in cell fate 1 and 2, respectively (*q* < 0.01). (E) Gene expression patterns in cells with different fates. Dashed black line represents the best fit line for the expression levels of genes in cells associated with cell fate 1, and the solid line represents the best fit line for the expression levels of genes in cells associated with cell fate 2.

To explore the specific differentiation relationships of cochlear neural cells in *R. ferrumequinum* , pseudo‐time analysis was conducted on SGN1, SGN2 and glial cells. This analysis identified two distinct differentiation cell fates from glial cells to neural cells, termed cell fate 1 and cell fate 2, as illustrated in Figure 7A. Cells from Cluster 2 were primarily found at the beginning of the pre‐branch. After differentiation, cell fate 1 primarily included cell Clusters 18, 20, while cell fate 2 primarily included cell Clusters 0, 1. The pre‐branch primarily included cells from glial cells; cells from SGN1 were mainly found on the cell fate 2 branch, while cells from SGN2 were mainly found on the cell fate 1 branch. According to the gene expression patterns of cells undergoing different differentiation fates, a group of differentially expressed genes associated with the transition from glial cells to SGN1 and SGN2 was detected and then clustered into five distinct clusters based on their expression levels at different neural cells (Figure 7B). GO enrichment results have indicated that genes from Clusters 3 and 4, associated with the differentiation fate from glial cells to SGN2, were involved in key biological processes: nervous system development (GO: 0007399), neuron development (GO: 0048666), regulation of neuron projection development (GO: 0010975) and neuron differentiation (GO: 0030182) (Figure 7C). Genes from Clusters 1 and 5, associated with the differentiation fate from glial cells to SGN1, were involved in key biological processes, such as synaptic transmission (GO: 0007268), trans‐synaptic signalling (GO: 0099537) and synaptic signalling (GO: 0099536) (Figure 7D). These findings robustly imply the potential of glial cells to undergo differentiation into SGN cells. Furthermore, a group of key fate‐determining genes integral to the transition of glial cells into SGN2 phenotypes was identified, such as *SYN3*, *KCNQ5* and *TNN*, which exhibited a sustained upregulation pattern along the cell fate 1 differentiation trajectory (Figure 7E). In addition, a group of genes including *DLG1*, *PDE3A* and *RIMS1* displayed a sustained upregulation pattern throughout the cell fate 2 differentiation trajectory (Figure 7E). These results provided compelling evidence for the neuronal origin from glial cells in the mammalian cochlea and revealed divergent molecular pathways in the differentiation of glial cells into SGN1 and SGN2, respectively.

## 4 Discussion

Single‐cell transcriptomics enables systematic characterisation of cellular diversity in the cochlea, shifting the paradigm of neuroscience research from the traditional focus on tissue anatomy to the molecular classification of cell types. Echoing early anatomical studies, sequencing bat cochlea using snRNA‐seq reveals a profound cellular diversity (Enge et al. 2017; Tabula Muris Consortium 2018; Han et al. 2018; Zeisel et al. 2018). Although single‐cell RNA sequencing studies have begun providing compendia of cell expression profiles, they have proven more difficult to systematically identify and localise all molecular types in specific organs to create a full molecular cell atlas (Travaglini et al. 2020). Herein, two comprehensive single‐cell atlases of the cochleae from *R. ferrumequinum* and *M. pilosus* bats were obtained. These atlases have uncovered the diversity of cochlear cell types in two bat species, advancing our understanding of the critical cell types and molecular pathways involved in the intricate processing and recognition of sound stimuli within the cochleae of echolocating bats. Moreover, these two cochlear cell atlases could serve as invaluable resources for the adaptive evolution of high‐frequency hearing in echolocating bats. This study has provided hundreds of marker genes for dozens of specific cell types and facilitated an in‐depth investigation of the expression levels and dynamic changes of target genes within particular cell types. This will provide robust support for elucidating the cellular composition, function and gene regulatory mechanisms of the bat cochlea, propelling in‐depth development of related research. These two cochlear cell atlases of *R. ferrumequinum* and *M. pilosus* also enable us to initiate comparative analyses of bat cochlear cell types through phylogenetic integration with cell atlases from other mammal species. Such multi‐species single‐cell comparisons could offer systematic evidence for the evolutionary conservation of chiropteran cochlear cell types, including neurons, neurosensory epithelium cells and immune cells, among others.

In recent work by R. Benjamin on the evolution of inner ear neuroanatomy in bats and its implications for echolocation, it was demonstrated that a significant difference in the spiral ganglion structures exists between *Yinpterochiroptera* (mainly containing CF bats) and *Yangochiroptera* (mainly containing FM bats) (Sulser et al. 2022). The spiral ganglion in *Yinpterochiroptera* is encased in a protective Rosenthal's canal, while *Yangochiroptera* displays a wall‐less Rosenthal's canal. The architectural disparity, suggesting that FM bats have developed an enlarged spiral ganglion liberated from the constraints of bony canal porosity, allows for a larger ganglion with more neurons, higher innervation density and denser clustering of cochlear nerve fascicles. Similar results were found in this study; an obviously higher proportion of SGNs were detected in the cochleae of FM bats compared to CF bats, providing direct cellular‐level evidence for the conclusion derived from neuroanatomy as referred to above. Furthermore, *R. ferrumequinum*, a CF bat species, possesses more SGN2 subclusters compared to *M. pilosus* , a FM bat species, at any given resolution. This finding indicates a higher degree of SGN2 differentiation and diversity in *R. ferrumequinum* , supporting the presence of dense minute perforations on the walls of the Rosenthal's canal in CF bats. The unique anatomical structure of the ganglion may lead to the differences in the types of SGNs between these two bat species, which in turn results in distinct echolocation types and consequently different echolocation behavioural functions in bats. Moreover, cross‐species comparative analysis has revealed the presence of a unique class of osteoblasts in the cochlea of *R. ferrumequinum* compared to *M. pilosus* . Previous studies have demonstrated that several adaptively evolved genes were significantly enriched in osteoclast differentiation and ossification pathways in CF and Click bats, who possess the walls of the Rosenthal's canal (Wang et al. 2020). In addition, the *CAT* gene, which is under positive selection in CF bats and involved in ossification pathways, was also detected to be expressed in the osteoblast cells of *R. ferrumequinum* . We suppose that the adaptively evolved genes in CF and click bats, together with key genes in osteoblasts (Table S6), collectively facilitated the formation of the Rosenthal's canal wall. These further suggest that the unique ganglion anatomical structure and the diversity of SGNs may underlie the specific CF echolocation types for bats from *Yinpterochiroptera*.

Taking the cochlear cell atlases as reference, we integrated snRNA‐seq and bulk RNA‐seq data to identify genes highly expressed in the cochleae of CF bats which were associated with high‐frequency hearing. These genes were predominantly detected in neuronal cells (SGN1 and SGN2), neurosensory epithelium cells and the lateral wall, indicating that adaptive changes in the auditory perception mechanism of CF bats were primarily observed in these cell types. In the mammalian cochlea, the onset of neuronal activity results from coordinated signalling from hair cells, supporting cells and SGNs. More specifically, cochlear hair cells are depolarised upon deflection of their stereocilia, which triggers the release of glutamate from hair cells. Glutamate binds to synaptic receptors on adjacent SGNs, resulting in the generation of action potentials and transmission of the afferent signal to the auditory brainstem. Further analysis, particularly the analysis of cell–cell interactions in *R. ferrumequinum* , has uncovered a specific receptor‐ligand interaction between SGNs and hair cells, namely Nrg1 and Erbb4. This interaction is pivotal for the generation of action potentials, ensuring the efficient transmission of auditory signals within the nervous system. The lateral wall, primarily responsible for the generation and maintenance of the endocochlear potential (EP), also plays a crucial role in regulating cochlear ion homeostasis and in the formation of the blood‐labyrinthine barrier (BLB) (Johns et al. 2023). Hair cells rely on a highly positive EP for appropriate mechanotransduction, which results in the release of neurotransmitter and the propagation of neural signalling to the cochlear afferent neurons via the spiral ganglion cells. In the cochlea of bats, the macroscopic specialisations of the basilar membrane, tectorial membrane and spiral ligament influence the wave propagation along the basilar membrane to a certain extent, giving rise to the auditory fovea (Schnitzler and Denzinger 2011). It is the synergistic action of neuronal cells, lateral wall cells and neurosensory epithelium cells that is adapted to high‐frequency hearing and Doppler shift compensation mechanisms of CF bats. Highly expressed genes associated with nervous system activities, detected in the cochleae of CF bats by comparative transcriptome, are predominantly localised to neural cells. However, the proportion of cochlear SGNs in CF bats is not larger than FM bats, suggesting the existence of differences in SGN types between these two bat species. Based on current knowledge of cochlear cell classification, SGNs in *R. ferrumequinum* , *M. pilosus* and also rodents are traditionally considered to belong to a single category. Nevertheless, cross‐species analysis confirmed the differences in SGN1 between the two bat species, with the presence of a specialised type of neuron (SGN1) in CF bats. An in‐depth analysis of the gene composition, functional differences and expression patterns of this specialised SGN1 has revealed distinct expression patterns between these two bat species, especially with significant differences in upregulated genes. For the species‐specific SGN1 of *R. ferrumequinum* , pathways were notably enriched, including those involved in glutamatergic synapse, dopaminergic synapse and GABAergic synapse, as well as the calcium signalling pathway and synaptic vesicle cycle. For the upregulated genes in *M. pilosus* specific SGN1 significantly enriched in pathways, including Amyotrophic lateral sclerosis, spinocerebellar ataxia and the glutamatergic synapse. SGNs in CF and FM bats may involve different physiological processes and behaviours through specific sets of cells and also upregulated genes.

Notably, in *R. ferrumequinum* bat‐specific SGN1, the key signalling pathway of GABAergic synapse is further related to GABA_A_ and GABA_B_ receptors, which play a pivotal role in neurotransmitter transmission. Previous studies have demonstrated that frequency tuning of auditory‐sensitive neurons sharpens progressively from the periphery to the central auditory system, a refinement primarily attributed to the action of various inhibitory neurotransmitters, with GABAergic neurotransmission playing a predominant role (Smotherman et al. 2003). A previous study of *R. ferrumequinum* utilised GABA_A_ agonists and antagonists in neurophysiological experiments and discovered the modulation of call frequency and Doppler shift compensation could be regulated through the GABA_A_ receptors (Firzlaff and Schuller 2001; Winer et al. 2011). This study indicated that the pathways and potential functions associated with highly expressed genes in cochlear neural cells were adapted to Doppler shift compensation of CF bats. At the cellular level, we have found the existence of a class of cochlear neurons with highly refined frequency tuning in the auditory fovea of *R. ferrumequinum* , known as SGN1, which could modulate Doppler shift compensation through the GABAergic synapse pathway via GABA_A_ and GABA_B_ receptors. Furthermore, another key signalling pathway in the species‐specific neurons, the calcium signalling pathway, plays a crucial role in regulating the development and survival of auditory neurosensory epithelium cells. The coordinated signal transduction between neurons and neurosensory epithelium cells represents an adaptive strategy to cope with the high‐frequency auditory requirement in CF bats.

Furthermore, the subclusters of cochlear SGN1 from *R. ferrumequinum* and *M. pilosus* indicated that, at the same resolution, *R. ferrumequinum* exhibited fewer subclusters compared to *M. pilosus* (as shown in Figure 3G), suggesting a relatively lower diversity in neural differentiation and a more uniform neural phenotype in *R. ferrumequinum* . We suppose that this uniformity in neural characteristics may be the adaptation to the narrow frequency band for the CF bats' vocalisation, inferring a highly precise frequency tuning of neurons to respond to the CF component, closely related to the narrow bandwidth of their echolocation calls. Although CF bats have a more homogeneous SGN1 type, genes associated with neural activity were expressed at higher levels in CF bats compared to FM bats, maintaining the sensitivity of neural cells to the amplitude and frequency modulations produced by the flapping wings of insects, enabling precise perception and processing of the CF signal. In the study of *Pteronotus parnellii* , a bat species capable of Doppler shift compensation, often referred to as a high‐duty‐cycle bat, researchers observed that its large spiral limbus structure appears to be specialised for processing the constant frequency component (CF component) within harmonic sound waves. In our study, comparative analysis of cochlear cell types between the two bat species revealed that spiral limbus cells are predominantly present in the cochlear single‐cell atlas of *R. ferrumequinum* . These findings may reflect, at the cellular level, the specialised response of the spiral limbus to specific CF components, thereby enhancing our understanding of sound wave processing and perception in bat echolocation mechanisms. These discoveries not only reveal the central role of neuronal cells, neurosensory epithelium cells and the spiral limbus (a category of lateral wall cells) in the Doppler shift compensation mechanism of CF bats, but also provide important theoretical foundations and cellular bases for subsequent research on Doppler shift compensation.

The cochlear neural cells in bats play an indispensable role in the auditory system. Notably, as a unique group of mammals, bats exhibit distinctive adaptations in the structure and function of their auditory nervous system, with heightened sensitivity and precision compared to other animals, particularly in the localisation and identification of high‐frequency signals. This superior capability is partly attributed to the specialised structure and function of the cochlear neural cells within their auditory apparatus. Following neural death in the retina, glia could serve as neural precursors for regenerated retinal neurons. In this study, we found that in the cochlea of *R. ferrumequinum* , glial cells expressed marker genes characteristic of neural progenitor cells, suggesting that these glial cells may possess the latent capacity of progenitors to regulate the migration, maturation and survival of spiral ganglion neurons (SGNs). Neurons also express NRG, which binds to ErbB receptors located on the astrocyte, an interaction necessary for normal glial morphology and neuronal migration (Monzack and Cunningham 2013). In the cochlear cell interaction network of *R. ferrumequinum* , we indeed identified a functional relationship between Nrg1 and ErbB4. Notably, genes highly expressed in glial cells were significantly enriched in the ErbB signalling pathway, which pathway could activate downstream PI3K/Akt signalling through molecules such as ErbB4, thereby modulating the phosphorylation status of key proteins and influencing cellular differentiation processes, then promoting the proliferation of progenitor cells while inhibiting their differentiation into specific cell types (Jiang et al. 2023). Additionally, we found interactions between glial cells and neurons mediated by Ncam1–Ncam1 and Ncam1–Ncam2. Researchers have identified NCAM as a key neuronal cell adhesion molecule that plays a pivotal role in neuron–neuron and neuron–glial adhesion processes (Brand et al. 2015). It is implicated in the regulation of various cellular processes within both the central and peripheral nervous systems, encompassing axonal cone growth, axonal pathfinding, myelination, fasciculation of nerve fibres and cell migration. We found significant up‐regulation of Ncam1 and Ncam2 genes in SGNs and glial cells in the cochlea of *R. ferrumequinum*. All of the above findings collectively suggested the presence of intercellular interactions between glial cells and SGNs within the cochlea of *R. ferrumequinum*. Furthermore, our study indicated that glial cells may act as progenitor cells within the cochlea, supporting neuronal function and survival in complex ways. Further results from pseudo‐time analysis of SGNs and glial cells have revealed that SGNs originate from glial cells, and a series of key differentiation genes have been identified. This will not only help us gain a deeper understanding of the developmental mechanisms and regulatory networks of the cochlear nervous system but also provide insights into the adaptive changes in the auditory nervous system of bats over the course of their evolution and also the comparative neurological differences with other types of bats and mammals.

To date, our understanding of cochlear cell types in other mammals remains limited. Most studies have focused on model organisms such as humans and mice, primarily driven by efforts to elucidate the genetic basis of sensorineural deafness. Previous studies have characterised the transcriptomic profiles of nearly all cochlear cell types in humans and mice, including circulating cells (mostly blood cells) and resident cochlear cells (spiral ganglion cells, glial cells, supporting cells, hair cells and cells from surrounding structures, etc.). Overall, the cochlear cell types detected here in the two bat species are similar to those in other mammals, reflecting the conservation of the mammalian auditory system. However, certain cell types (e.g., neural cells) and structures show unique differences, likely reflecting the specific auditory requirements and ecological adaptations specific to echolocating bats.

## 5 Conclusion

In this study, we constructed a comprehensive cochlear single‐cell atlas for two typical echolocating bats, integrating histology, cell biology and molecular biology to systematically reveal the spatial positioning, cellular composition and molecular differences between the cochleae of CF and FM bats. This work advances our understanding of the critical cell types involved in the intricate analysis and recognition of sound waves within the cochlea, a key auditory organ for echolocating bats. Results of multi‐species single‐cell comparative analysis provided systematic evidence for the evolutionary conservation of cochlear cell type programmes in chiropterans, such as neuronal cells, neurosensory epithelium cells and immune cells. Moreover, this study has deepened our understanding of the Doppler shift compensation mechanism in CF bats, offering new insights for research in the field of neuroscience. By uncovering key regulatory genes that govern the differentiation of glial cells into SGNs, this study provided novel clues for understanding the development and function of the bat cochlear nervous system. New findings here could contribute to the advancement of bat behavioural ecology, neuroscience and cell biology, facilitating interdisciplinary progress in these fields.

## Author Contributions

X.W. and H.W. conceived and designed the study, analysed the data with help from all authors and wrote the manuscript. M.B., R.S., W.D., Y.Z., Y.P., A.L. and J.L. performed the sampling and assisted with the interpretation of the data. H.W. provided supervision during the entire project. J.F. and K.S. assisted with study design and provided laboratory space and reagents. All authors have read and approved the final article.

## Ethics Statement

The animal study was reviewed and approved by the Laboratory Animal Welfare and Ethics Committee of Jilin Agricultural University (approval code: 20210607001). All efforts were made to minimise the suffering of the animals.

## Conflicts of Interest

The authors declare no conflicts of interest.

## Supporting information


**Figure S1.** Quality control of snRNA‐seq.

**Figure S2.** WGCNA clustering analysis of two species.

**Figure S3.** The top 10 KEGG pathways with the smallest *p* values, enriched by upregulated genes in each cell group of *R. ferrumequinum* (see Table S2 for details).

**Figure S4.** t‐SNE plot showing the differently expressed gene *Fgf14, Kcnip4, Rbfox1, KIRREL3* in all cells.

**Figure S5.** UMAP analysis of SGN2 cell type heterogeneity in *R. ferrumequinum* and *M. pilosus* at varied resolutions.

**Figure S6.** Sankey diagram comparing Cell subtypes assignments for *R. ferrumequinum* and *M. pilosus* .

**Table S1.** Detailed information of marker genes used for cell type identification.
**Table S2.** KEGG pathways significantly enriched by upregulated genes in each of the five cochlear cell groups of *R. ferrumequinum* .
**Table S3.** Cell types corresponding to the 26 cell clusters obtained by cross‐species analysis of *R. ferrumequinum* and *M. pilosus* .
**Table S4.** The conserved cell gene expression matrix based on homologous genes from two bat species.
**Table S5.** GO terms significantly enriched by upregulated genes in SGN1 of *R. ferrumequinum* .
**Table S6.** Upregulated genes detected in osteoblasts of *R. ferrumequinum* .

## Data Availability

The authors confirm that the data supporting the findings of this study are available within the article and its Supporting Information.
