# Supplementary figures and images for: Cochlear Cell Atlas of Two Laryngeal Echolocating Bats—New Evidence for the Adaptive Nervous Physiology in Constant Frequency Bat
Source: Mol Ecol Resour. 2025 Mar 20;25(6):e14101. doi: 10.1111/1755-0998.14101 (PMC12225709; doi:10.1111/1755-0998.14101)

A

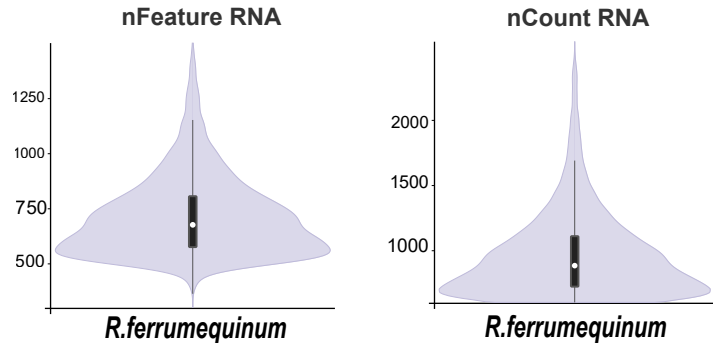

B

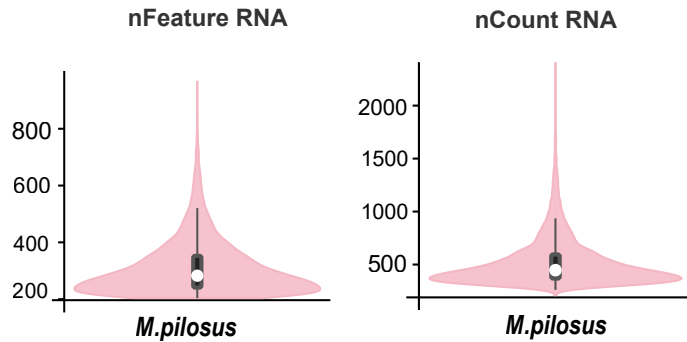

C

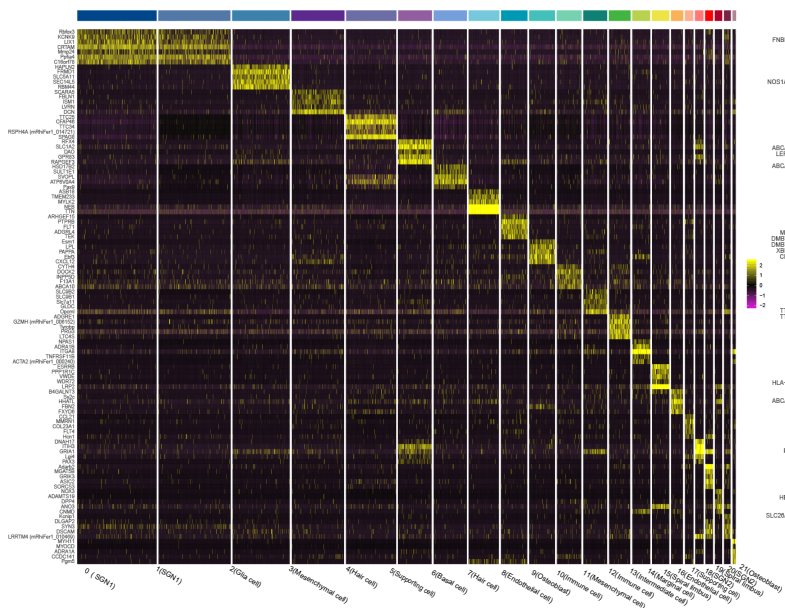

D

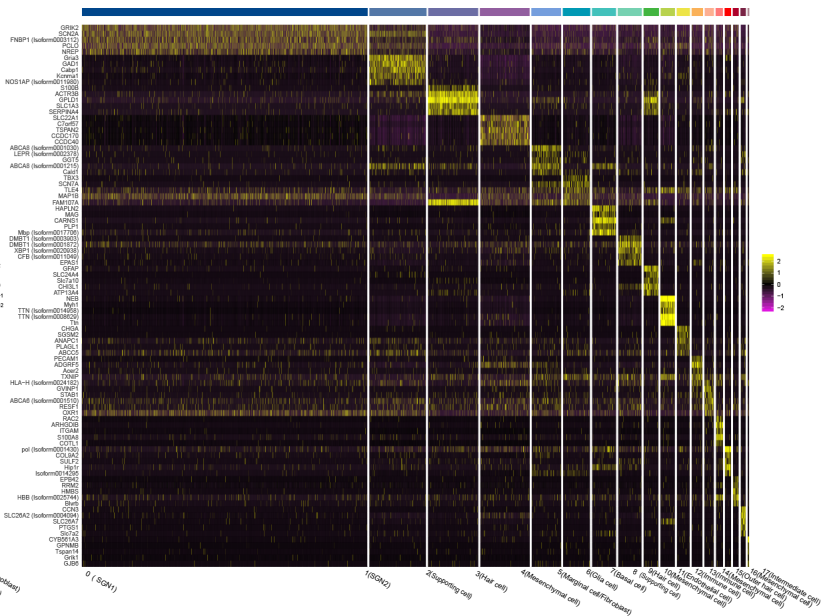

Supplement: Supplementary file 1 — Figure S1. Quality control of snRNA‐seq. [file MEN-25-e14101-s004.pdf]

**A**

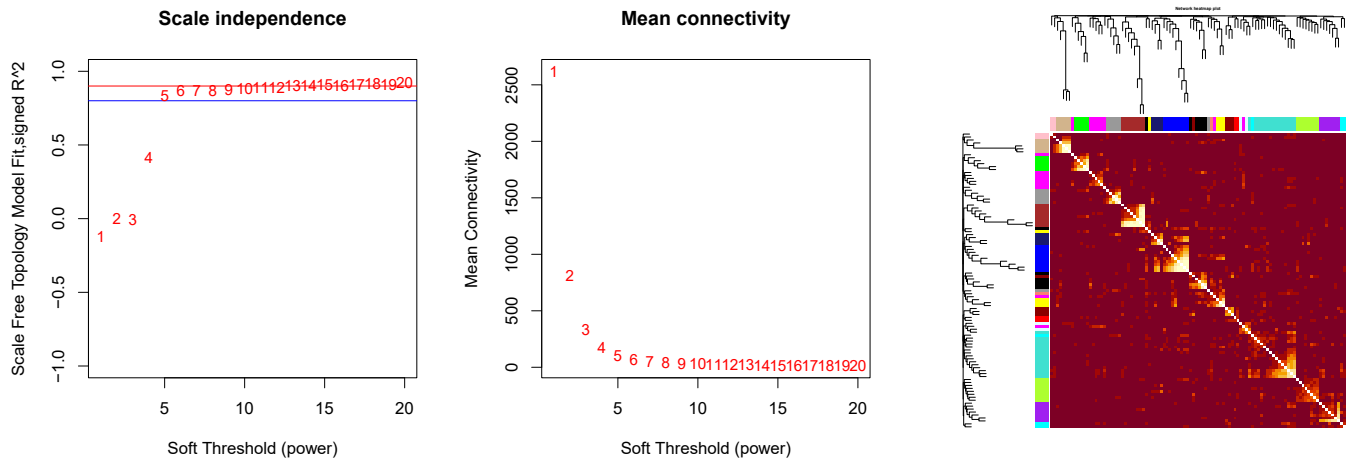

**B**

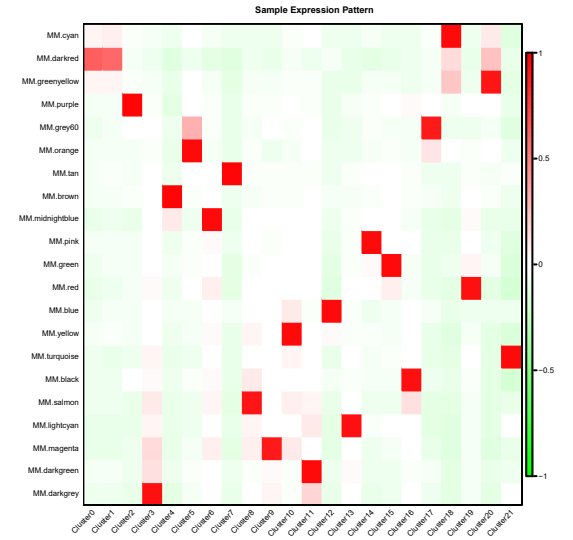

**C**

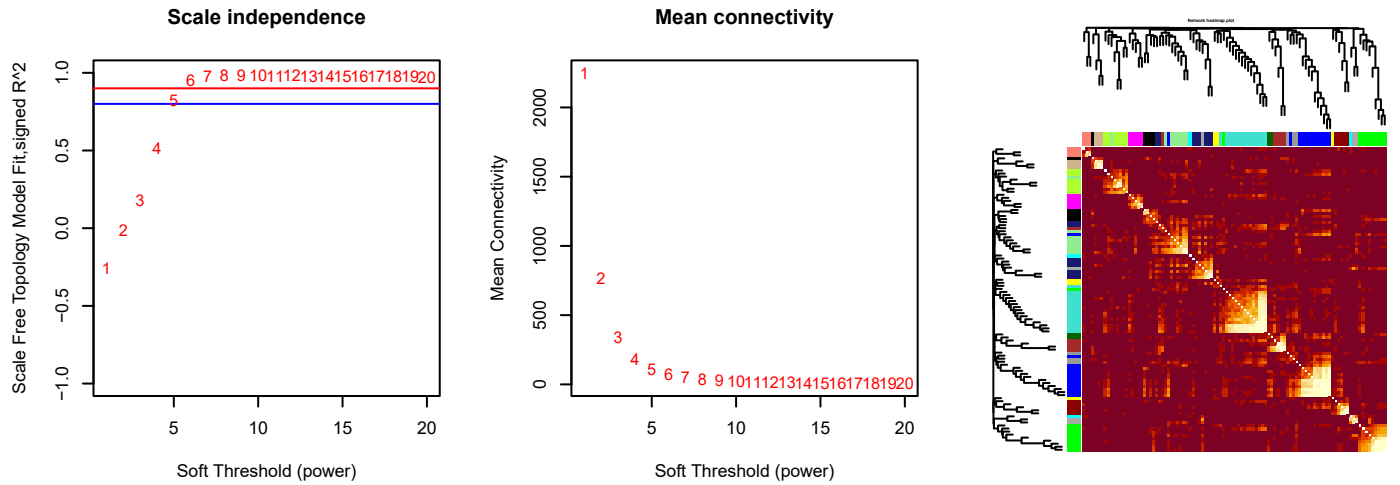

**D**

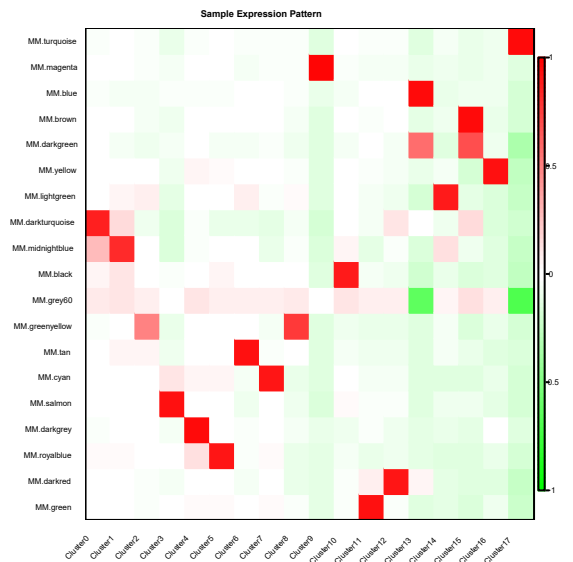

Supplement: Supplementary file 2 — Figure S2. WGCNA clustering analysis of two species. [file MEN-25-e14101-s003.pdf]

## Neural cell

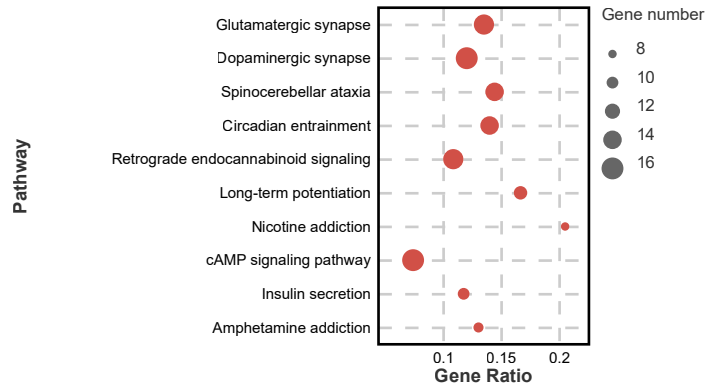

## Neurosensory epithelium

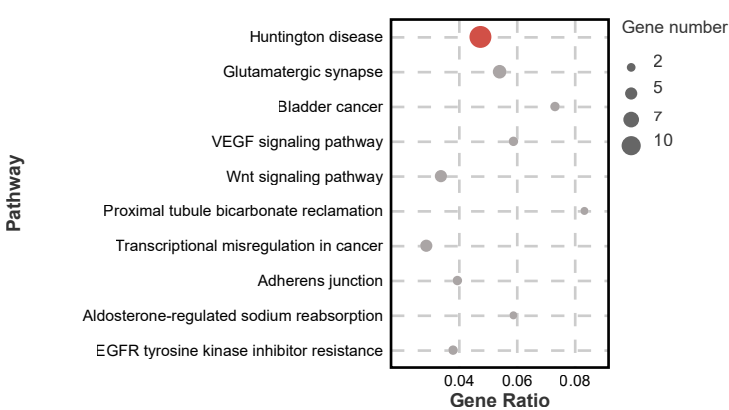

## Immune cell

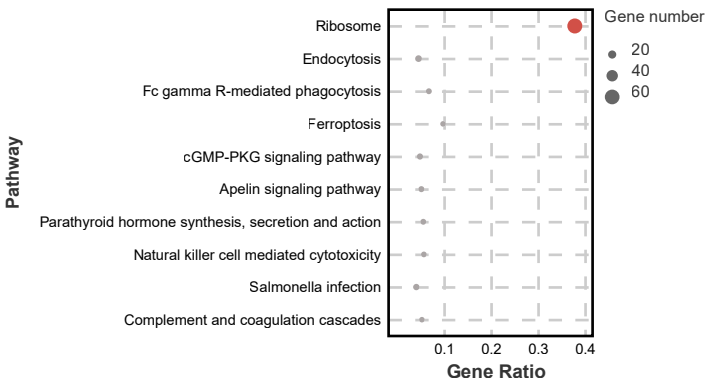

## Surrounding structure

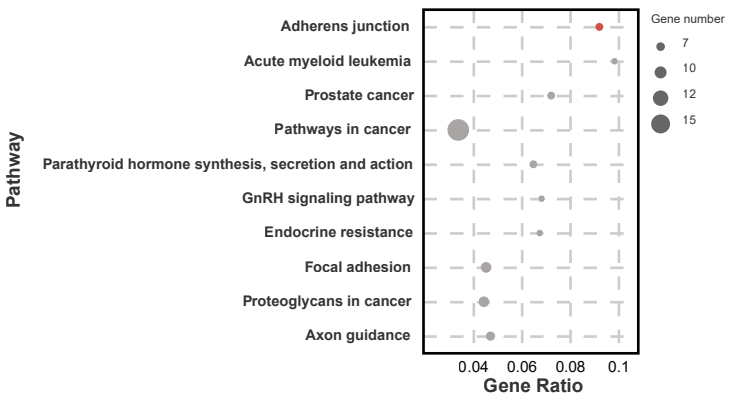

## Lateral wall

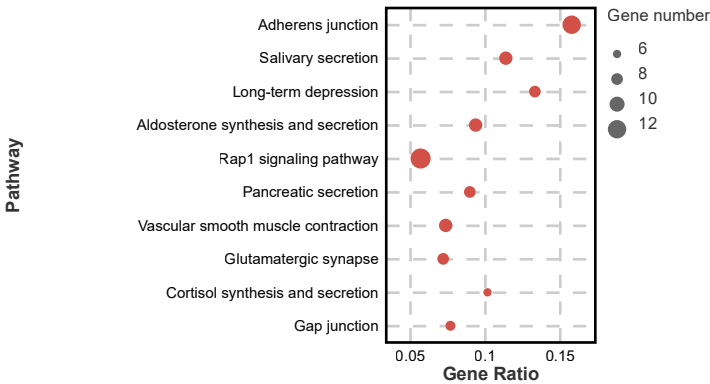

Supplement: Supplementary file 3 — Figure S3. The top 10 KEGG pathways with the smallest p values, enriched by upregulated genes in each cell group of R. ferrumequinum (see Table S2 for details). [file MEN-25-e14101-s006.pdf]

***Fgf14***

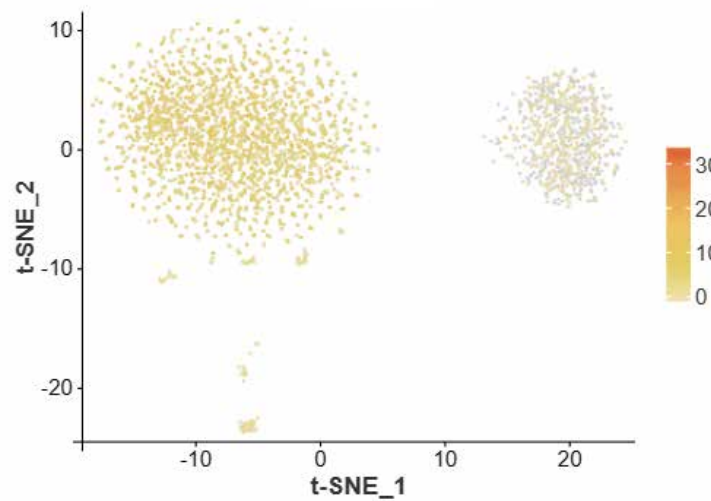

***KCNIP4***

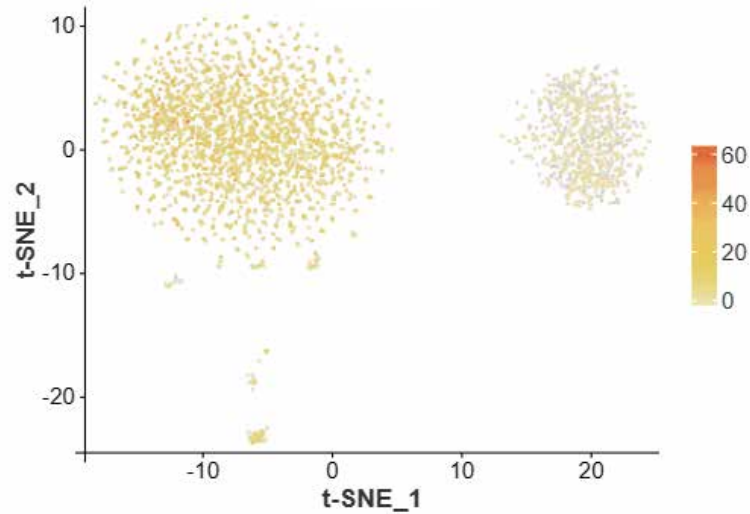

***RBFOX1***

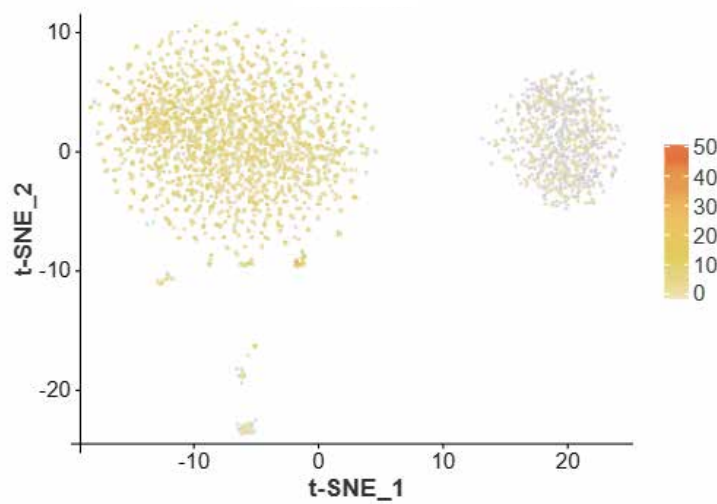

***KIRREL3***

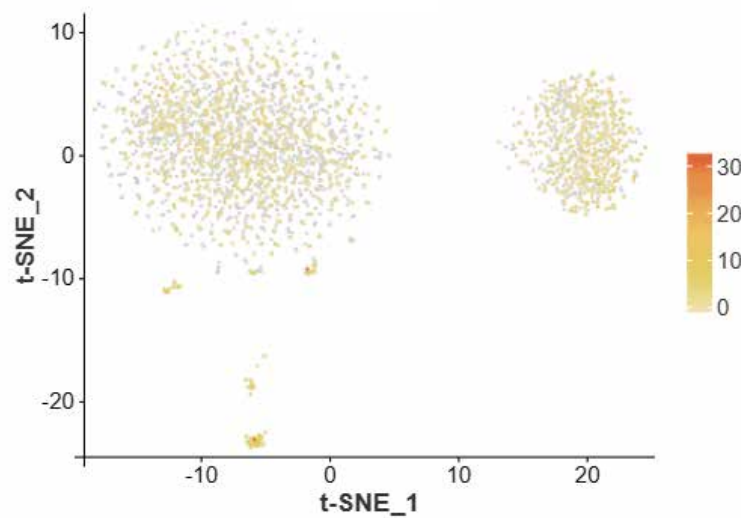

Supplement: Supplementary file 4 — Figure S4. t‐SNE plot showing the differently expressed gene Fgf14, Kcnip4, Rbfox1, KIRREL3 in all cells. [file MEN-25-e14101-s007.pdf]

*R. ferrumequinum*

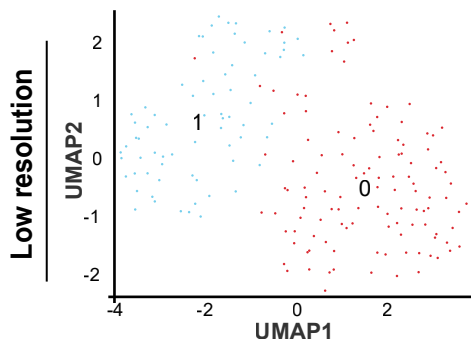

*M. pilosus*

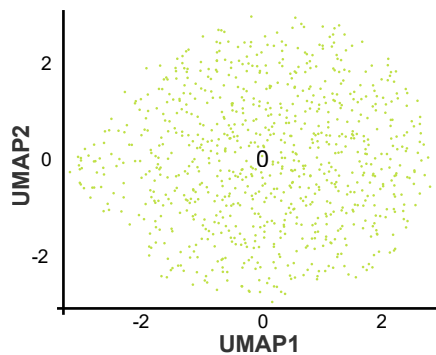

High resolution

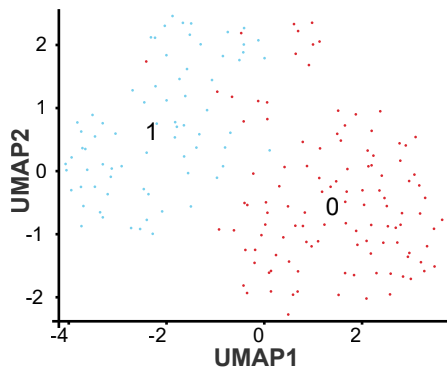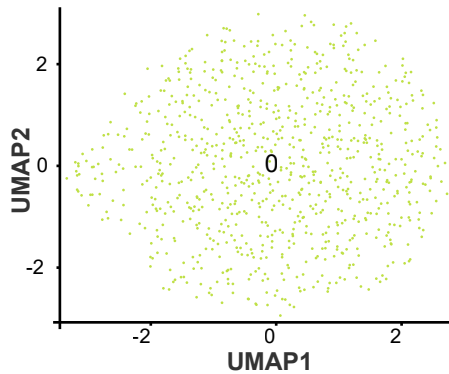

Supplement: Supplementary file 5 — Figure S5. UMAP analysis of SGN2 cell type heterogeneity in R. ferrumequinum and M. pilosus at varied resolutions. [file MEN-25-e14101-s005.pdf]

Neurosensory epithelium

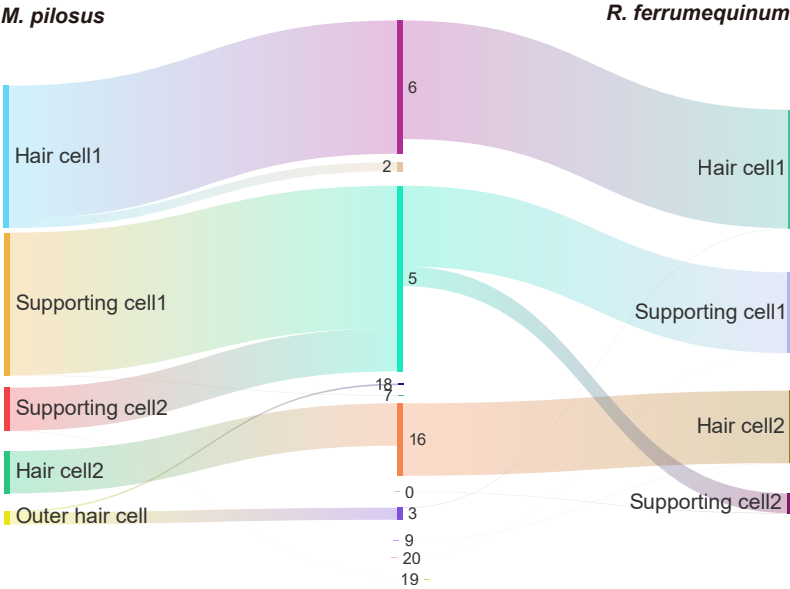

Immune cell

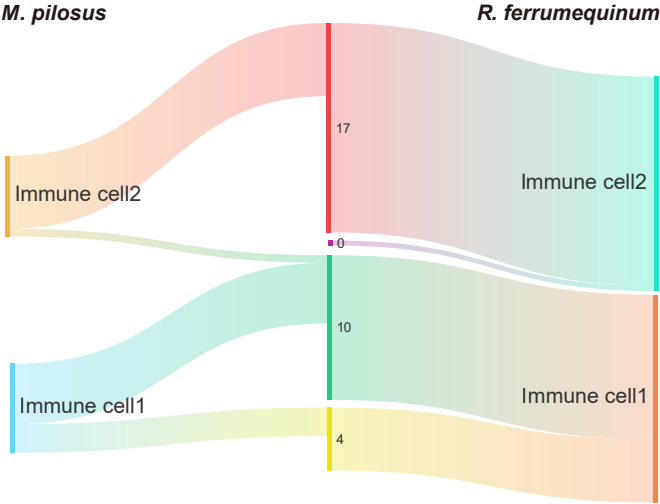

Lateral wall

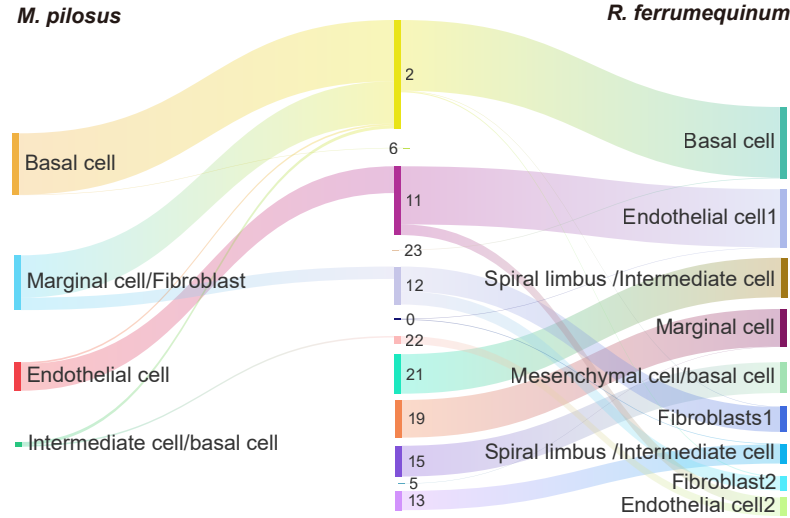

Surrounding structure

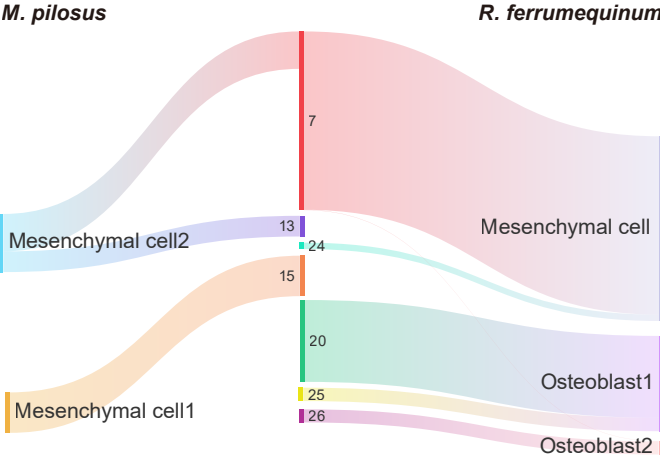

Neuronal cell

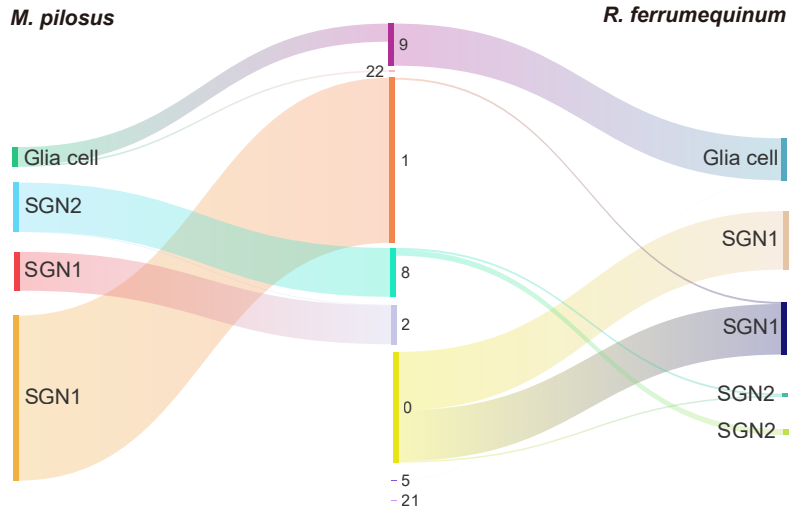

Supplement: Supplementary file 6 — Figure S6. Sankey diagram comparing Cell subtypes assignments for R. ferrumequinum and M. pilosus . [file MEN-25-e14101-s002.pdf]
